# Questions Asked by Students during a Course of Lectures on *the Organon*

**Published:** 1889-05

**Authors:** 

**Affiliations:** Chicago


### HOMŒOPATHIC PHYSICIAN,

A MONTHLY JOURNAL OF

HOMŒOPATHIC MATERIA MEDICA AND CLINICAL MEDICINE.

"If our school ever give up the strict inductive method of Hahnemann, we are lost, and deserve only to be mentioned as a caricature in the history of medicine."—CONSTANTINE HERING.

Vol. IX. MAY, 1889. No. 5.

### QUESTIONS ASKED BY STUDENTS DURING THE COURSE OF LECTURES ON THE *ORGANON* AS GIVEN BY PROF. GEE, OF CHICAGO.

1. How does a drug cure by the law of similars? Why does it not increase the disease?

The process of restoration as brought about by the curative remedy is largely a matter of conjecture. Some theories have been advanced as to how it *may* be effected; as, for instance, by a change of polarity and repulsion resulting as with two negatives or two positives.

Hahnemann has given his theory in page 29 of the *Organon:* "* * * * somewhat stronger, similar, artificial morbid affection is implanted upon the vital power, deranged by a natural disease, * * * * but owing to its brief duration it will soon be overcome by the vital force, which, liberated first from the natural disease, and finally from the substituted artificial [drug] affection, now again finds itself enabled to continue the life of the organism in health."

He gives illustrations in the succeeding sections, but states an important fact in Section 28: "A scientific explanation of *its mode of action* is of little importance; I therefore place but a slight value upon an attempt at explanation."

2. When two remedies are markedly indicated, do you prescribe the one covering the greater number of symptoms, or the one covering the symptoms giving the greater distress to the patient?

This is a practical question, and the answer to it must be conditional. Ordinarily we should give the remedy covering the greater number of symptoms. Quality of symptoms must be considered. A remedy may meet a large number of common or unimportant symptoms, and not be the curative one.

The symptoms giving most distress to the patient must be carefully studied *and interpreted* in making the prescription. The more the nervous system is disturbed by them, if *peculiar*, the more likely are they to be guiding symptoms.

Section 153 covers this point very well: "The more *prominent*, *uncommon*, and *peculiar* [characteristic] features of the case are especially, and almost exclusively considered and noted; for *these in particular should bear the closest similitude to the symptoms of the desired medicine*, if that is to accomplish the cure. The more general and indefinite symptoms, etc., deserve but little notice, etc." (Read it all carefully, and refer to it every week or oftener, especially if you have a troublesome case on hand.)

The difficult part is in taking the case. Number of symptoms cannot make up for quality. The key is the *interpretation* of the symptoms.

Remember the common ones in making the so-called "diagnosis."

3. When one remedy does not cover all the symptoms, and there is another remedy which will exactly cover the balance of them, why not mix the two drugs, if they do not act chemically on each other?

You are not likely to meet a case where a remedy will cover "all the symptoms;" in fact, too close similitude would lead us to suspect poisoning by that remedy. Nor is it necessary that the remedy should meet all of them. The *guiding* symptoms are the ones which should bear the closest similitude.

We do not know when a remedy will affect the human organism curatively until it has been "proved."

We must, then, give the remedy on those indications, and without interference, to expect curative results.

Give the remedy indicated by most of the guiding symptoms, give it time to act, and then take the case anew.

4. If we know our materia medica well, how can we help but associate remedies with the various symptoms as they are derived from the patient?

This is one great cause of failure, for the student who knows a great amount of materia medica is greatly tempted to prescribe hastily and spoil the case or make a failure. He does not wait to take the case fully, but frequently interrupts the patient when a characteristic is touched, in trying to fit the patient to the remedy, instead of taking all the symptoms and suiting the remedy to the totality.

5. Is not clinical experience just as fallacious and liable to change in the hands of the homœopathic physician as in the hands of the allopath?

Most certainly. The experience varies constantly. No two are alike. No two cases of the same disease are alike. Our experience is helpful to us in many ways. A better acquaintance with remedies enables us to more readily distinguish the individuality of each, as the close acquaintance of a friend enables us to recognize him at a distance, in a crowd, and with exactness.

